# Dr. S. A. Sudderth

**Published:** 1859-04

**Authors:** 


Obituary.—It is with regret we learn that Dr. S. A. Sudderth, a graduate of the Baltimore College of Dental Surgery, died at his residence, Morgantown, N. C., on the 26th of December, 1858. Dr. S. was a member of the M. E. Church, at the time of his decease.

